# Culture shock among international students in Turkey: an analysis of the effects of self-efficacy, life satisfaction and socio-cultural adaptation on culture shock

**DOI:** 10.1186/s40359-024-01641-9

**Published:** 2024-03-15

**Authors:** Mustafa Almukdad, Engin Karadag

**Affiliations:** 1 Akdeniz University, 07070 Antalya, Turkey; 2 Belvedere International School, Abu Dhabi, United Arab Emirates

**Keywords:** International student, Culture shock, Self-efficacy, Life satisfaction, Sociocultural adaptation

## Abstract

In this study, we investigated whether the interaction effects of self-efficacy, life satisfaction, and sociocultural adjustment have significant negative effects on culture shock. The data were collected from 323 international students in Turkey, and the sample comprised 197 undergraduates (61%) and 126 graduates (39%). We administered the “Culture Shock Questionnaire,” “General Self-Efficacy Scale”, “The Satisfaction with Life Scale”, and “Sociocultural Adaptation Scale” through an online survey. Of the participants, 86 were female (26.6%), and 237 were male (73.4%). The ages of the participants ranged from 18 to 32 (*M* = 22.11; *SD* = 4.23). In this study, we identified three structural models to test the relationships between culture shock, self-efficacy, life satisfaction, and sociocultural adjustment. The results supported our hypothesis (H1) that there would be a significant negative relationship between self-efficacy and culture shock. One-way MANOVA revealed that students with lower self-efficacy scores experienced greater culture shock. Conversely, students with higher self-efficacy scores experienced lower culture shock and interpersonal stress. Additionally, a multigroup analysis was conducted to test the assumed relationships in structural models for Muslim (such as Turkey, where the research was conducted, which is 90% Muslim) and Christian students. The results indicated that self-efficacy has a significantly greater negative impact on culture shock for Christian students than for Muslim students. Our study confirmed the hypothesis (H4) that life satisfaction and sociocultural adjustment serve as mediating variables between self-efficacy and culture shock. Both life satisfaction and sociocultural adjustment were found to have significant direct impacts on culture shock, and a meaningful mediating effect on the relationship between self-efficacy and culture shock was identified. Based on these findings, we concluded that self-efficacy may be particularly beneficial for coping with culture shock for people who do not embrace dominant religious beliefs in a local culture.

## Introduction

The world is becoming increasingly more dimensional, both culturally and linguistically. People move across borders from one country to another. Higher education is one of the areas where such mobility is most intense. Studying abroad has become increasingly popular worldwide in recent years [1]. Today, more than 1 million international students on average enroll in higher education institutions each year outside their native countries. The data for 2019 indicate that the United States ranks first in this respect, with more than 1.2 million international students [3]. It is followed by the United Kingdom, with more than half a million international students, and by China, with more than 450 thousand students [4]. In recent years, international students have preferred alternative countries, including the USA and the UK, due to the demand above student capacity, high tuition fees, and high immigration barriers at popular destinations for higher education. For instance, students are increasingly beginning to move to countries such as New Zealand, South Korea, China, Turkey, and the Netherlands to pursue higher education.

Turkey is a European country with a large higher education capacity, with 203 universities, approximately eight million undergraduate students and approximately 200 thousand faculty members [5]. There were 48 thousand international students in Turkey in 2014. This number has increased to 180 thousand in recent years, mainly due to the target-oriented internationalization policy implemented by Turkey’s Higher Education Council (YOK) and the “Turkey Scholarships” launched in 2012 for international students, as well as due to Turkey’s geopolitical position, multicultural society, and high development levels [6]. The majority of the international students attending universities in Turkey are from Asian countries. In addition, there are also large portions of students from the Middle East and Arab majority countries. There are more international students in China and African countries. According to data on international students in 2019 [5], international students were mostly from the following five countries: Syria, Azerbaijan, Turkmenistan, Iran, and Afghanistan.

International students in Turkey are from 180 different countries. These students create a complex and culturally diversified society due to their differential cultural values, mental perspectives, and social norms. These students came from various countries with distinct cultural, social, and educational structures. There is a lack of studies dealing with how these materials can be successfully integrated into Turkey. To fill this gap in the related literature, this study aimed to examine the culture shock experienced by international students at a state university in Antalya and the factors affecting the severity of this cultural shock.

## Theoretical background and hypotheses

International students face great challenges as they leave their familiar world behind and try to adjust to a new physical, cultural, and linguistic location [7]. Due to difficulties in communication, lack of necessary social support and disturbed culture, international students most often experience deep isolation [8] and intense depression [9]. Furthermore, most international students experience a great deal of culture shock [1]. The term culture shock is generally attributed to Kalervo Oberg, who made it popular in his 1954 publication “*Culture Shock*”. Oberg [10] defines culture as an emotional condition “precipitated by the anxiety that results from losing all our familiar signs and symbols of social intercourse.” Based on Oberg’s assumption, Adler [11] considered culture shock “primarily a set of emotional reactions to the loss of perceptual reinforcements from one’s own culture, to new cultural stimuli which have little or no meaning, and to the misunderstanding of new and diverse experiences”. Recent studies provide the following definition for culture shock: “anxiety and a sense of confusion and uncertainty affecting a person when s/he is exposed to a new culture or environment without adequate preparation.” Culture shock is a phenomenological experience that is encountered since individuals themselves cannot use known and familiar cultural references in such an encounter [12]. Research suggests that culture shock has negative effects on international students’ psychological and sociocultural adaptation to their host environment [13, 14]. Given that culture shock has such adverse effects, it seems useful to reveal those factors that may reduce or eliminate culture shock among international students.

Contact with an unfamiliar culture can reportedly lead to anxiety, stress, mental illness, and physical illness among individuals [10]. Oberg [10] proposed six major dimensions of culture shock in his study: tensions due to intense adaptation attempts; feelings of loss and deprivation related to friends, status, profession, and property; feelings that the new culture is rejected by the host culture members or that the new culture rejects them; rolls, feelings of confusion in regard to values and self-identity; feelings of anxiety and even anger; and feelings of hopelessness about unfamiliar practices. As a result of these negative feelings, individuals cannot manage to deal with the new environment. However, not all individuals experience culture shock. However, experiencing culture shock depends on many factors, including the severity of individuals’ reaction to a new culture, the degree of control over this reaction, and certain interpersonal, biological, interpersonal, spatial-temporal, and geopolitical factors [15]. The following factors are reported to have effects on culture shock: lack of accommodation and transportation, higher living expenses, language barriers, religious differences, and separation from family members [13, 16].

International students who visit a different country for higher education may experience a greater level of culture shock as they age. As Lord and Dawson [17] suggested, international students can begin to experience tension upon their arrival at the airport and on their travel to the city where the university is located. They become particularly anxious and stressed about the road from the airport to the university. Being excited, depressed, self-doubt and lacking self-esteem are the typical emotions experienced in the first few weeks as individuals struggle to understand themselves in a new environment [18]. In general, most international students experience culture shock at certain levels. Culture shocks can begin on campuses even before trips and can be intensified during the first few weeks or months following their arrival [13]. They also exhibit several symptoms of culture shock, which are classified as psychological or physical cues. Some examples of these symptoms include despair, boredom, lack of concentration, aggressiveness, health-care problems, feelings of insecurity, anger, hopelessness, sleeping too much or getting tired easily, delays in daily routines and other minor disappointments such as irritation, suffering from body aches and pains, and longing to return home [12, 15, 19].

International students may encounter several difficulties during their studies in a foreign environment, even though they also face many opportunities or positive things. It is not surprising that there are many complex challenges faced by international students, especially if the host country’s culture is strikingly different from their own [20]. It has been reported that international students experience certain difficulties in the USA, such as academic difficulties, social isolation and cultural adaptation [21]. Similarly, Le, LaCost and Wismer [22] reported two major difficulties for international students: language-related problems and culture shock. Mclachlan and Justice [23] argue that international students are subject to mental health problem risks due to the lack of support systems and culturalization stress. Cultural shocks experienced by international students can be associated with variables related to individual differences [24]. There is a limited body of literature in higher education addressing the potential predictors of culture shock among international students, with existing studies predominantly focusing on variables such as mental health [83], social identity [84], coping strategies [85], and self-esteem [86]. Therefore, this study focused on determining the potential predictors of international students’ experiences of cultural shocks, and self-efficacy, life satisfaction, sociocultural adjustment and religious belief were selected as relevant individual difference variables.

### Self-efficacy

Bandura [25, 26] defined self-efficacy as ‘the beliefs of individuals in regard to their skills and abilities in achieving certain tasks using their present.” Bandura [25] described two major dimensions of self-efficacy: outcome expectations and competence expectations. He further argues that outcome expectations are based on individuals’ expectations about the outcome of an action while carrying out this action. The competence expectation is related to the consistency between an individual’s effort to act and his or her beliefs about his or her own individual competencies. Individuals evaluate the competence of their actions throughout their life and compare their actions with the actions of other people. An individual who believes that s/he has an ability in regard to any subject can develop a positive sense of self-efficacy even if s/he is not talented at all. The opposite situation is also possible. In other words, even if individuals have necessary levels of any ability, they may develop a negative sense of self-efficacy and tend to exhibit ineffective behaviors in this regard. Bandura [25] states that individuals with higher levels of perceived self-efficacy are more successful at controlling their environment and overcoming the difficulties they encounter. Another type of competence developed by Bandura [26] is outcome effectiveness. This type of competence refers to the ability of individuals to achieve a result by controlling environmental factors to achieve their goals.

Self-efficacy has a very significant role in education. There are studies dealing with the effects of international students’ self-efficacy in their adaptation to college or university environments [27, 28]. In the past twenty years, self-efficacy has been considered to be a significant predictor of students’ motivation and learning. It is suggested that for students with higher levels of self-efficacy beliefs, their beliefs function as a mediator of their academic success [29]. More specifically, international students with higher levels of self-efficacy are determined to be good at learning and imitating appropriate behaviors in the host country [30]. A robust sense of self-efficacy has been observed to be associated with better health, greater achievement, and increased social integration [87, 88, 89]. This implies that individuals with high self-efficacy are likely to experience less culture shock. Additionally, many studies have indicated that self-efficacy is closely connected with successful intercultural adaptation [31–33] and learning about the host culture [23]. Therefore, self-efficacy is assumed to play a role in shaping the culture shock experienced by international students, which produced the following hypothesis: **[H1]** There is a significant negative relationship between self-efficacy and culture shock. While there are numerous types of self-efficacy in the literature (such as academic self-efficacy), the majority of these thoughts are rooted in Bandura’s [25] theory of self-efficacy. Therefore, in our study, we found that it is accurate to utilize general self-efficacy, which forms the foundation of Bandura’s self-efficacy theory.

### Life satisfaction

Life satisfaction is an indicator of psychological adjustment and includes individuals’ satisfaction with their current, past, and future life [34]. Life satisfaction allows individuals to have healthy, successful, rich, and good social relationships. These are desired points by everyone but may differ from one person to another. Life satisfaction is an area of positive psychology that analyses individuals’ cognitive and affective evaluations of their lives [35]. Appleton and Song [36] suggested that life satisfaction has six different components. These variables are as follows: [1] income, [2] occupational and social status, [3] potential and social mobility, [4] wealth-related conditions, [5] current state policies and [6] environmental, familial, and social relations. It is argued that life satisfaction is a dynamic pattern that can change based on individuals’ current living conditions and personal standards [37]. Research on life satisfaction indicates that factors such as the number of friends, financial satisfaction, perceived discrimination, and the information received before arrival significantly affect the life satisfaction of international students. Language proficiency (based on the host’s native language and English language) and having a roommate have a significant positive effect on life satisfaction [38]. The results of a study on a sample of international students in the USA indicate that their psychological problems decrease as the duration of stay in the host country increases [39]. Therefore, it is assumed that individuals’ life satisfaction shapes their culture shock experiences. Based on this assumption, the second hypothesis of the study, **[H2]**, is stated as follows: There is a significant negative relationship between life satisfaction and culture shock.

### Sociocultural adaptation

In an attempt to integrate concepts into a fractionated area of research, Ward and his colleagues proposed that cross-cultural adaptation may be meaningfully divided into two domains: psychological (emotional/affective) and sociocultural (behavioral). The former refers to psychological wellbeing or satisfaction; the latter is related to the ability to “fit in,” to acquire culturally appropriate skills and to negotiate interactive aspects of the host environment [40]. Accordingly, Ward [41] has argued that psychological adjustment can best be understood in terms of the stress and coping framework, while sociocultural adaptation is best explained by social skills or a culture learning paradigm.

Sociocultural adaptation is defined in terms of behavioral competency. It is strongly influenced by factors that form the basis of cultural learning and social skills acquisition. These factors include the duration of stay in the new culture, cultural knowledge, the amount of interaction and identification with the citizens of the host country, cultural distance, language fluency and acculturation strategies [40, 42, 43, 44]. In brief, sociocultural adaptation is understandable within the social learning paradigm and refers to how well an individual who is acculturated can manage daily life in his or her new cultural context [45].

In addition, sociocultural adaptation includes the adaptation of younger students who need to adapt to school and be successful at their schools, as well as the adaptation of adults who must fulfill the requirements of their profession and work. Therefore, it is assumed that sociocultural adaptation has a shaping effect on culture shock. Based on this assumption, the third hypothesis of the study, **[H3]**, is stated as follows: There is a negative relationship between sociocultural adaptation and culture shock.

Finally, it should be added that it is very difficult to adapt to a new cultural environment. Addressing culture shock requires a positive attitude toward the values and acts of a different cultural setting. When the cultural adaptation process is analyzed in terms of the values of students, fewer conflicts allow them to easily adapt to social and academic environments [16]. Strong self-efficacy feelings are closely related to much better health conditions, greater achievement and greater social integration [46, 47, 48]. Therefore, the last hypothesis of the study, namely, **[H4]**, is stated as follows: Given that self-efficacy, life satisfaction, sociocultural adaptation and culture shock are interrelated, life satisfaction and sociocultural adaptation are mediator variables between self-efficacy and culture shock.

## Method

### Sample

Due to the ambiguity in the literature and in Turkey regarding the definitions of international and foreign students, we initially established a definition for international students in our study: “students who venture beyond national or regional boundaries for the purpose of education and are located outside their country of citizenship” [90]. We subsequently determined the scope of our study. The population of our study comprises 2,549 international students enrolled in public universities in Turkey [5]. In our study, we did not employ any sampling method or communicate with all students in the population (for details, refer to the “Procedures” section). A total of 323 undergraduate and graduate international students attending a public university in Turkey participated in the study. The data of the study were collected through an online survey questionnaire. Of the 323 participants, 86 were female (26.6%), and 237 were male (73.4%). It clearly shows that the international students in Turkey are mostly male, as indicated in the related data previously [5]. The age of the participants varied between 18 and 32 years (*M* = 22.11; *SD* = 4.23). Table 1 presents the demographic backgrounds of the teachers constituting the research sample.

**Table 1:** Distributions of the sample with respect to demographic background

Variable								
Gender		Male	Female					
	*n*	237	86					
	%	73.4	26.6					
Degrees		BA	MA	PhD				
	*n*	197	68	58				
	%	60.9	21.1	18.0				
		Engineering and Architecture	Management and Business	Social and Humanities	Medical Sciences	Science		
	n	139,0	75,0	46,0	39,0	18,0		
	%	43,85	23,66	14,51	12,30	5,68		
Religious Beliefs		Muslims	Christians	Other				
	*n*	261	44	14				
	%	81.8	13.8	4.4				
Countries		Middle East	Sub-Saharan African	European	Turkic Republics (of the former Soviet Union)	North Africa	Southern Asia	Far East
	*n*	101	66	47	34	33	19	15
	%	32.1	20.9	14.9	10.8	10.5	6.0	4.8

The study was carried out in Antalya, which is situated on the Mediterranean coast and is Turkey’s fifth largest city as well as Turkey’s largest tourism center; this city is one of the world’s leading tourism centers. There are more than 100 thousand foreigners residing in cities in more than 100 different countries [49].

### Instruments

#### The culture shock questionnaire (CSQ)

The CSQ comprises two subscales and twelve items (namely, ‘Core’ Culture Shock and Interpersonal Stress) [50]. The subscales are *Core Culture Shock* (“Do you feel strain from the effort to adapt to a new culture?”, an exemplary statement from this subscale) and *Interpersonal Stress* (“Do you feel anxious or awkward when meeting local people?”, an exemplary statement from this subscale?”. A higher score on the CSQ indicates a greater level of culture shock. The answers to the items are given on a 5-point Likert-type scale ranging from 1 (never) to 5 (always). The internal consistency coefficients varied between 0.71 and 0.81 (Table 2).

**Table 2:** Descriptive statistics, correlation coefficients and study variable reliability

	α	M	SD	1	1a	1b	2	3	4
**1-**CSQ	0.80 [0.78,0.84]	2.22 [2.16,2.46]	0.61 [0.59,0.71]	-					
**1a-**CCS	0.76 [0.74,0.80]	2.26 [2.20,2.53]	0.61 [0.67,0.74]	0.95 [0.95,0.94]	-				
**1b-**IS	0.82 [0.81,0.85]	2.12 [2.07,2.31]	0.71 [0.67,0.86]	0.73 [0.70,0.80 ]	0.48[0.44,0.58]	-			
**2-**GSE	0.84 [0.83,0.88]	3.02 [3.03,2.97]	0.52 [0.51,0.53]	-41. [-0.36,-0.56]	− 0.37[-0.33,-0.51]	− 0.34[-0.28,-0.51]	-		
**3-**SCAS	0.92 [0.91,0.90]	2.98 [3.03,2.68]	0.55 [0.54,0.55]	− 0.64 [-0.63,-0.59]	− 0.62[-0.60,-0.58]	− 0.44[-0.43,-0.45]	0.22[0.26,0.27]	-	
**4-**SWLS	0.73 [0.70,0.84]	2.93 [2.95,2.89]	0.72 [0.69,0.78]	− 0.36 [-0.33,-0.46 ]	− 0.33[-0.30,-0.42]	− 0.30[-0.27,-0.39]	0.36[0.43,0.14]	0.24[0.19,41]	-

Note: CSQ: The Culture Shock Questionnaire; CCS: Core Culture Shock; IS: Interpersonal Stress; GSE: General Self-Efficacy Scale; SCAS, Sociocultural Adaptation Scale; SWLS: The Satisfaction with Life Scale, total [Muslim students, Christian students]

#### General self-efficacy scale (GSE)

The GSE comprises ten items and one dimension. The GSE aims to measure individuals’ belief in their ability to cope with stressful and difficult life events [52] and their general confidence in regard to new situations that are difficult to cope with or unfamiliar to individuals [53]. A higher score indicates a higher level of self-efficacy. The answers to the items are given on a 4-point Likert-type scale ranging from 1 (not at all true) to 4 (exactly true). The two sample items from the GSE are “I can always manage to solve difficult problems if I try hard enough” and “If someone opposes me, I can find the means and ways to get what I want”. In the present study, the internal consistency coefficient of the GSE was found to be 0.84 (Table 2).

#### Sociocultural adaptation scale (SCAS)

The SCAS aims to measure the challenges faced by newcomers in meeting their daily needs, establishing meaningful relationships with the host society, and measuring their understanding of the values of the host culture [90]. High scores on the SCAS indicate low difficulty in social fields and higher levels of sociocultural adaptation. The answers to the items are given on a 5-point Likert-type scale ranging from 1 (extremely difficult) to 5 (not difficult). The items “Making friends”, “Finding food you enjoy”, “Communicating with people of a different ethnic group” and “Understanding the local political system” are four sample items from the SCAS. In the present study, the internal consistency coefficient of the SCAS was 0.88 (Table 2).

#### The satisfaction with Life Scale (SWLS)

The SWLS addresses a cognitive/judgmental process and aims to measure the quality of life of individuals based on the criteria they choose. For the SWLS, high scores indicate higher levels of life satisfaction [91]. The answers to the items are given on a 5-point Likert-type scale ranging from 1 (strongly disagree) to 5 (strongly agree). “My life is close to ideal in many ways”, “my life conditions are perfect” and “I’m satisfied with my life” are three sample items from the SWLS. In the present study, the internal consistency coefficient of the SWLS was 0.83 (Table 2).

### Procedure

At the beginning of the study, the Human Subjects Ethics Committee approved the study protocol. An online research package encompassing demographic questions and items from the CSQ, GSE, SCAS, and SWLS was created, and communication was initiated with international students. Subsequently, emails were sent to all international students (*n* = 2549) at public universities inviting them to participate in the study. The invitations included information on the purpose of the study, brief details about the data collection instruments, ethical procedures, the voluntary nature of participation, and the absence of any rewards or compensation. Those who voluntarily expressed their willingness to participate in the study (response rate 13%) were first briefed on the study’s objectives, signed informed consent forms were collected, and participants were provided with information regarding the confidentiality, voluntariness, and anonymity of their participation. When they confirmed their participation, a link was sent to them, and they were asked to join the study.

**Fig. 1:**
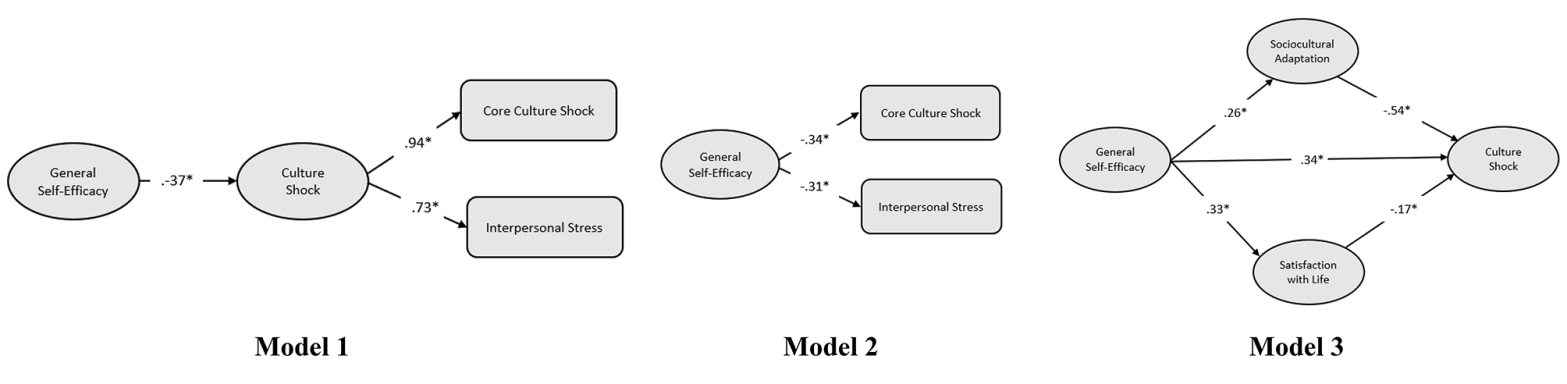
Competing structural models

Multigroup structural equation modeling (SEM) on LISREL (ver. 8.51) was employed to test the direct effects of self-efficacy and the indirect effects of life satisfaction and sociocultural adaptation on culture shock for Muslim and Christian students (in Turkey, the percentage of Muslims was 98% in 2012) [54] and 89% in 2019) [55]. In brief, structural models were designed to analyze the connections between religious beliefs and other variables. To test the mediation of Model 3 shown in Fig. 1, an analysis was performed using the PROCESS macro via SPSS [56]. According to the model, sociocultural adaptation and life satisfaction are the mediators of the relationship between self-efficacy and culture shock. Culture shock was the dependent variable (Fig. 1). The analysis revealed the following: [1] the effects of self-efficacy on culture shock (both indirect and direct effects through sociocultural adaptation and life satisfaction), [2] the effects of sociocultural adaptation on culture shock and [3] the effects of life satisfaction on culture shock (Model 4 by Hayes). The statistical significance of the direct and indirect effects was evaluated by means of 5,000 bootstrap samples to create bias-corrected confidence intervals (CIs; 95%) with heteroscedasticity consistency. According to the data analyses carried out in the present study, the significance criterion was set at *p < *.05. As a final step, a confirmatory factor analysis (CFA) was performed to test whether the models were proposed and whether the measurement models fit the data.

**Table 3:** The multi-group path analysis results

Model	Path	Estimate	standardizing η	*t*-value	*p*-value
**Model 1**	GSE → CSQ	− 0.37 (-0.83)	− 0.32 (-0.62)	-5.24 (-5.07)	< 0.01 (< 0.01)
**Model 2**	GSE → CCS	− 0.39 (-0.86)	− 0.30 (-0.62)	-5.01 (-5.10)	< 0.01 (< 0.01)
	GSE → IS	− 0.34 (-0.76)	− 0.26 (-0.47)	-4.22 (-3.47)	< 0.01 (< 0.01)
**Model 3**	GSE → SWLS	0.26 (0.46)	0.25 (0.44)	4.12 (3.21)	< 0.01 (< 0.01)
	GSE → SWLS	0.56 (0.48)	0.41 (0.33)	7.14 (2.28)	< 0.01 (< 0.01)
	GSE → CSQ	− 0.12 (-0.50)	− 0.11 (-0.38)	-1.99 (-3.23)	< 0.01 (< 0.01)
	SWLS → CSQ	− 0.58 (-0.46)	− 0.54 (-0.36)	− 0.10.85 (-3.20)	< 0.01 (< 0.01)
	SWLS →CSQ	− 0.16 (-0.24)	− 0.19 (-0.26)	-3.55 (-2.44)	< 0.01 (< 0.01)

*Note*: CSQ: The Culture Shock Questionnaire, CCS: Core Culture Shock, IS: Interpersonal Stress, GSE: General Self-Efficacy Scale, SCAS, Sociocultural Adaptation Scale, SWLS: The Satisfaction with Life Scale, Muslim students [Christian students]

## Results

### Instrument validity and reliability

Before the analyses, a confirmatory factor analysis was performed to evaluate the construct validity of the scales. Before the analysis of the results obtained, it was determined whether the values predicted exceeded the theoretical limits. The values did not exceed the limits. The chi-square (χ2) value and the statistical significance level related to the confirmatory factor analysis were identified. Depending on the degree of freedom, the low chi-square (1.3) value suggested that the proposed model was suitable for analyzing the collected data. In addition, other goodness-of-fit indices of the models showed that the model proposed for the measurement models was suitable (*see*. Table 4). These results confirm that the modeled factor structures are confirmed when the values obtained for the measurement models within the scope of the standard fit indices are examined. The standardized coefficients obtained through the confirmatory factor analysis, which indicate the correlations between factors and items, were found to vary between 0.37 and 0.77. The results suggest that the measurement tools used in the study are adequate and therefore provide evidence for construct validity. The Cronbach’s α coefficients varied between 0.70 and 0.90 (Table 2), indicating that the internal consistency was between good and very good.

### Common method bias

To test for common method bias, Harman’s one factor test was employed [57]. All the dependent and independent variables were examined through confirmatory factor analysis, and the factors accounted for 68.01% of the total variance. However, the first factor accounts for only 22.70% of the total variance. These findings indicate that there is no common method bias in the dataset [58].

### Descriptive findings

The culture shock levels, self-efficacy, sociocultural adaptation and life satisfaction of the international students who participated in the study are described in Table 2. The level of culture shock among international students was at the medium-low level (*M* = 2.22, *SD* = 0.61). The scores for both core culture shock (*M* = 2.26, *SD* = 0.61) and interpersonal stress (*M* = 2.12, *SD* = 0.71) were found to be at the medium-low level. The participants’ scores for life satisfaction (*M* = 2.93, *SD* = 0.72) and sociocultural adaptation (*M* = 2.98, *SD* = 0.55) were at the medium levels, and their self-efficacy scores (*M* = 3.02, *SD* = 0.52) were found to be at the medium-high level. Before testing the theoretical models developed in the present study, the correlation coefficients for the correlations among culture shock scores, self-efficacy scores, sociocultural adaptation scores and life satisfaction scores were examined (Table 2). The results indicate that there is a significant negative correlation between the total score and both the subscale scores of culture shock and the scores of self-efficacy, sociocultural adaptation and life satisfaction. In addition, positive significant correlations were found between self-efficacy and life satisfaction and between self-efficacy and adaptation to sociocultural processes.

### Structural model

Multigroup SEM analysis was used via maximum likelihood to explore hypothesized relationships in the theoretical models for Muslim and Christian students, as shown in Fig. 1. Model 1 suggests that self-efficacy has a negative and significant impact on culture shock (*r*=-.37, *t*=-7.18, *R*^2^ = 0.14, *p <*.001). Model 2 also suggested that self-efficacy has a negative and significant impact on the following dimensions of culture shock: ‘core culture shock’ (*r*=-.34, *t*=-6.49, *R*^2^ = 0.12, *p <*.01) and ‘interpersonal stress’ (*r*=-.31, t=-6.49, R^2^ =.10, *p <*.01). Model 3 indicates that self-efficacy has positive and significant effects on sociocultural adaptation (*r* =.26, *t* = 4.85, *R*^2^ =.07, *p <*.001) and life satisfaction (*r* =.33, *t* = 6.35, *R*^2^ =.11, *p <*.001). Additionally, both sociocultural adaptation (*r*=-.54, *t*=-12.47, *R*^2^ =.29, *p <*.001) and life satisfaction (*r*=-.17, *t*=-.39, *R*^2^ =.03, *p <*.001) had negative and significant effects on culture shocks. These results indicate that self-efficacy, sociocultural adaptation, and satisfaction with life in the prediction of culture shock are significantly different from zero at the.001 level. Therefore, H1, H3 and H4 were supported. The results of the multigroup analysis performed for Models 1, 2 and 3 are shown in Table 3.

**Table 4:** Multiple mediation analysis

Effect	Coeff	SE	*t*	*p*	LLCI	ULCI
General self-efficacy on satisfaction with life	0.29	0.05	5.08	0.001	0.17	0.40
General self-efficacy on sociocultural adaptation	0.50	0.07	7.03	0.001	0.36	0.64
Satisfaction with life on culture shock	− 0.13	0.03	-3.59	0.001	− 0.20	− 0.06
Sociocultural adaptation on culture shock	− 0.58	0.04	-12.82	0.001	− 0.67	− 0.49
General self-efficacy on culture shock	− 0.24	0.05	-4.69	0.001	− 0.34	− 0.13
Direct effect	− 0.24	0.05	-4.69	0.001	− 0.34	− 0.13
Indirect effect (via the satisfaction with life)	− 0.06	0.02	-	-	− 0.15	− 0.11
Indirect effect (via the sociocultural adaptation)	− 0.17	0.03	-	-	− 0.24	− 0.10

### Mediation analysis

Given that, in Model 3, the mediating effects of life satisfaction and adaptation to sociocultural processes did not include zero within the 95% CI range, the multiple mediation index was significant (*Effect*_*Satisfaction with life*_=-0.06, 95% CI [.-15, − 0.11]; *Effect*_*sociocultural adaptation*_=-0.17, 95% CI [.-24, − 0.10]) (Hayes, 2013). This evidence indicates that the conceptual model is robust. Specifically, self-efficacy led to greater satisfaction with life (*Effect* = 0.29, *t* = 5.08, *p* <.001; 95% CI [0.17, 0.40]) and sociocultural adaptation (*Effect* = 0.50, *t* = 7.03, *p* <.001; 95% CI [0.36, 0.64]), providing support for Hypotheses 3 and 4. In line with Hypotheses 3 and 4, feelings of sociocultural adaptation (*Effect*=-0.58, *t*=-12.82, *p* <.001, 95% CI [-0.67, − 0.49]) and satisfaction with life (*Effect*=-0.13, *t*=-3.59, *p* =.001, 95% CI [-0.20, − 0.06]) were negatively related to students’ culture shock. In addition, a significant direct effect was found for self-efficacy in relation to culture shock (*Effect*=-0.24, *t*=-4.59, *p* =.001, 95% CI [-0.34, − 0.13]). The Sobel test indicated that the direct mediating effects of self-efficacy on culture shock (in other words, both life satisfaction and adaptation to sociocultural processes) were statistically significant (*p <*.01). These results support the mediating effects of self-efficacy and show that self-efficacy has significant effects on culture shock through life satisfaction and adaptation to sociocultural processes (the results of the PROCESS macro are summarized in Table 4). Therefore, H6 was supported.

### Model estimation

Table 5 summarizes the results of the fit indices. In Model 1, a hidden factor was found, which was reflected in two variables related to culture shock: [1] ‘core culture shock’ and [2] ‘interpersonal stresses’. In Model 2, the direct effects of self-efficacy on general culture shock were examined. In Model 3, the indirect effect of self-efficacy on general culture shock through adaptation to sociocultural processes and life satisfaction was tested. Comparative and residual-based fit indices collectively show that the models proposed are suitable for both religious beliefs and for both Muslim and non-Muslim groups. These findings suggest that all three models have higher prediction levels and can be employed to determine the predictive value of self-efficacy for GS. On the other hand, the difference in the chi-square test nestedness between models was found to be statistically significant (*p* <.001).

**Table 5:** Fit indices of the measurement and theoretical models

Model	CQS	GSE	SCAS	SWLS	Model 1	Model 2	Model 3	Acceptable Values
*p* value	0.001	0.001	0.001	0.37	0.001[0.001,0.001]	0.001[0.001,0.001]	0.003[0.16,0.03]	> 0.05
*X*^2^/df	1.9	2.1	2.5	1.0	1.3[1.9,1.7]	1.1 [1.8,1.6]	1.6[1.8,1.7]	< 3 (Kline, 2005)
RMSEA	0.07	0.07	0.07	0.02	0.05[0.07,0.07]	0.04[0.06,0.07]	0.05[0.06,0.05]	< 0.08 (Hu & Bentler, 1999)
GFI	0.91	0.90	0.90	0.98	0.92[0.91,0.91]	0.94[0.93,0.92]	0.99[0.98,0.98]	≥ 0.90 (Kline, 2005)
AGFI	0.89	0.85	0.89	0.95	0.91[0.90,0.91]	0.92[0.91,0.90]	0.91[0.93,0.92]	≥ 0.85 (Cole, 1987)
NFI	0.90	0.90	0.91	0.97	0.93[0.92,0.93]	0.92[0.94,0.91]	0.97[0.99,0.98]	≥ 0.90 (Hu & Bentler, 1999)
CFI	0.95	0.94	0.92	0.99	0.95[0.94,0.93]	0.96[0.97,0.95]	0.87[0.90,0.91]	≥ 0.90 (Hu & Bentler, 1999)

*Note*: CSQ: Culture Shock Questionnaire; CSCAS: Sociocultural Adaptation Scale; SWLS: Satisfaction with Life Scale; total [Muslim students, Christian students]

### Randomization tests

The analyses in the study were carried out using the data obtained from a nonrandom sample. Randomization tests were performed to support the generalizability of the findings beyond the research sample. In addition, 5000 bootstrap replicates were used to test the effects of self-efficacy on culture shock through sociocultural adaptation and life satisfaction. The analysis of the averages, standard errors, 95% CIs, significance levels and aspects of relationships indicates that the bootstrapped samples are close to each other.

### Summary of the results

Hypotheses 1–4 were confirmed and accepted by the findings. In this study, the self-efficacy, sociocultural adaptation, and life satisfaction of international students in Turkey were analyzed. It was found that higher levels of all these variables help reduce the cultural shock that international students experience. However, self-efficacy was found to have a particularly significant impact on culture shock both directly and indirectly through life satisfaction and sociocultural adaptation.

## Discussion and conclusion

In this study, we aimed to analyze the cultural shock of international students, which has been mostly neglected in studies on higher education [59, 60]. Moreover, to fill this gap, this study evaluated the effects of individuals’ self-efficacy, life satisfaction, and sociocultural adaptation on culture shocks on the basis of individuals’ religious beliefs. The models developed in the study are unique in regard to the use of theoretical structures, which is new to the samples of international students even though these models were developed before.

First, culture shock is inherently caused by stressful life changes. Therefore, people who engage in intercultural interactions need to be resilient, adapt, and develop coping strategies and tactics to address such new settings [61, 62, 63]. It can be argued that individuals with low self-efficacy experience greater culture shock. Therefore, these individuals are expected to be more prone to both lower success and the intention to leave. Therefore, a significant relationship was expected between self-efficacy and culture shock. The results revealed that there was a significant and negative relationship between self-efficacy and culture shock. As self-efficacy scores increase, individuals are found to have less of a tendency toward culture shock. In addition, participants with low self-efficacy scores were found to experience much greater interpersonal stress. On the other hand, participants with higher self-efficacy scores were found to experience less culture shock and interpersonal stress. These individuals are less willing to change their mental and behavioral structures and consequently to adapt successfully to their new environment [27, 63]. These findings suggest that self-efficacy is important for international students in dealing with culture shock. Ulusoy [64] argues that both cultural intelligence and self-efficacy are two major elements in understanding individuals from different cultures, having proper perceptions about them and managing cultural differences during cultural exchanges. These findings are consistent with the current findings on self-efficacy and culture shock [27, 31, 60, 65, 66, 67].

In this study, the role of religious beliefs was also examined concerning the correlation between self-efficacy and culture shock. The findings indicate that Muslim students and Christian students vary in terms of the culture shock they experience. Specifically, for students whose religious beliefs are different from those of the local community (Christian students), the correlations between self-efficacy and culture shock are much stronger than those for those whose religious beliefs are the same as those of the local community. In addition, Christian students experience much greater culture shocks. Therefore, it is reasonable to predict that having the same religious beliefs as locals produces lower levels of culture shock. In brief, the results suggest that self-efficacy and religious beliefs are key to culture shock, and students with different religious beliefs who are self-efficacious and have cultural backgrounds incompatible with the local culture experience stronger culture shock. This finding is compatible with previous findings [39, 60, 68, 69, 70]. Similarly, many studies [20, 71, 72] report that students coming from countries that are culturally close or similar to the host country experience less stress and difficulty adapting than students coming from culturally distant countries. Therefore, cultural shock prevention efforts should be considered throughout cultural orientations.

Previous studies have shown that there are correlations between self-efficacy, life satisfaction, sociocultural adaptation and culture shock [14, 28, 31, 60, 73]. However, the results of the present study provide the details of these relationships. Both life satisfaction and sociocultural adaptation have important mediating effects on the relationship between self-efficacy and culture shock. These results support the assumption that life satisfaction and sociocultural adaptation play important mediating roles in the relationship between self-efficacy and culture shock. It was also found that higher levels of self-efficacy produce greater life satisfaction and sociocultural adaptation, which further reduce culture shock. This is because higher levels of self-efficacy help students be more open to new experiences [74] and serve as an important preventive tool against stress and negativity that occur due to the unfamiliar foreign context [75]. This reduces loneliness, anxiety, and other maladaptive consequences [76]. The findings of the study support the findings reported by Edwards-Joseph and Baker [77] and Presbitero [14]. For this reason, knowing individuals’ religious beliefs, levels of life satisfaction and sociocultural adaptation can help individuals eliminate or at least reduce their culture shock.

### Managerial implications

The international student market in higher education has become a multibillion-dollar market in recent years. The related data for the period between 2008 and 2014 indicate that the countries with the highest income from international students include the United States, Australia, England, Canada, France, New Zealand and Germany [78]. This market has a particular position and ability to attract thousands of international students and is supported by many countries and higher education institutions. Decades of research and dozens of academics agree that one of the most important key variables in international students’ higher education experience is culture shock [28, 30, 79]. The findings of this study show that self-efficacy over culture shock is the key to culture shock, and higher levels of self-efficacy help individuals experience a very low level of culture shock, especially for students who do not have the same religious beliefs as those of the local community. In this context, consistent with the findings of many previous studies, it has been shown that students’ higher levels of self-efficacy while studying abroad are associated with greater life satisfaction and lower levels of culture shock [79, 80, 81].

Moreover, the findings show that when students develop higher levels of self-efficacy, these feelings also have a positive effect on their life satisfaction and adaptation to sociocultural processes, resulting in less cultural shock. This is a positive message for both students and administrators: Making students feel more competent has important consequences not only for students but also for education. Hence, higher education administrators should consider enhancing the self-efficacy of international students. This can be achieved, for example, by providing special guidance services for international students. In addition, the findings obtained from this study suggest that the greater the life satisfaction and sociocultural adjustment are, the stronger the correlation between self-efficacy and culture shock. Thus, the relationship between self-efficacy, life satisfaction, sociocultural adaptation and culture shock seems to be a more general concept for international students. Therefore, managers should constantly monitor the culture shock experiences of international students. In addition, Hsieh [82] suggested cultural education as a way to improve intercultural relations.

Finally, practitioners should be aware that high self-efficacy in itself is not enough to address culture shocks: sharing the same religious belief with the local community results in fewer negative experiences. Therefore, targeting only one group of students is not a necessary but rather a sufficient condition for a lower culture shock.

### Limitations and future research

Using a nonrandom sampling method in the selection of participants is one of the methodological limitations of the study. In addition, the cross-sectional nature of the research design prevented a deeper understanding of the relationships among self-efficacy, sociocultural adaptation, life satisfaction and culture shock. Therefore, it might be useful to conduct a longitudinal study using mixed methods approaches or experimental designs to investigate the subject in a more detailed way.

Another limitation of the current study is that concerning religious beliefs, the sects and living in accordance with religious beliefs are mostly ignored in the analyses. For some students, religious belief is a way of life, but for others, religious belief is a ritual or anything that has no significance. Therefore, scholars should be careful in generalizing these findings because the experiences may differ. Another limitation of the study is the city where the study was carried out: Antalya. Antalya is one of the leading tourism regions not only in Turkey but also in the world. This shows that the local community is accustomed to living with people of different races, from different countries and from different cultures. Therefore, the findings should be interpreted taking these points into consideration.

Although the study indicated the major effects of self-efficacy on culture shock, it did not address the reasons for culture shock concerning self-efficacy. Therefore, future studies may be designed as experimental studies to determine whether such effects are causal. On the other hand, even though there are a number of limitations in the present study, these findings are new contributions and new insights to the literature concerning this topic.

## Data Availability

The datasets generated and analyzed during the current study are available from the corresponding author on reasonable request.
